# Genomics Approaches to Deciphering Natural Transformation in Cyanobacteria

**DOI:** 10.3389/fmicb.2019.01259

**Published:** 2019-06-07

**Authors:** Kristen E. Wendt, Himadri B. Pakrasi

**Affiliations:** Department of Biology, Washington University in St. Louis, St. Louis, MO, United States

**Keywords:** cyanobacteria, transformation, competence, genomics, pilus, recombination

## Abstract

Natural transformation is the process by which bacteria actively take up and maintain extracellular DNA. This naturally occurring process is widely used as a genetic modification method in bacterial species, and is crucial for the efficient genetic modification of organisms in an industrial setting. Cyanobacteria are oxygenic photosynthetic microbes that are promising platforms for bioproduction of fuels, chemicals, and feedstocks. Using CO_2_ and sunlight alone, cyanobacteria can make these valuable bioproducts in a carbon-neutral manner. While genetic modifications have been performed in a number of cyanobacterial strains, natural transformation has been successfully demonstrated in only a handful of species. Even though thousands of cyanobacterial strains have been deposited in culture collections and hundreds of these species have had their genomes sequenced, only a few of these organisms have been experimentally transformed. Although there are many aspects of cyanobacterial biology that provide exciting opportunities for biological investigation, the absence of a rapid and straightforward genetic modification method such as natural transformation hinders research efforts to understand some of the fascinating nuances of cyanobacterial physiology. The ability to use natural transformation in more strains of cyanobacteria would facilitate the rapid employment of these organisms in bioproduction settings. This article discusses recent advances in the understanding of natural transformation in cyanobacteria. Additionally, it identifies gaps in the current knowledge about cyanobacterial natural transformation and provides an overview of how new genomic technologies may be implemented to understand this important process.

## Introduction

A handful of bacterial species are capable of natural transformation, a process by which bacteria actively take up and maintain extracellular DNA. This capacity plays an evolutionary role by providing a source for genetic diversity and serves as a valuable tool for genetic engineering in the laboratory. Cyanobacteria are a genus of oxygenic phototrophs in which natural transformation has been demonstrated in a few species. Although hundreds of species of cyanobacteria have been cultivated, only a few have been naturally transformed experimentally (Trehan and Sinah, 1981; Porter, 1986; Onai et al., 2004). Therefore, these select species have become widely used as model systems. However, numerous cyanobacterial species outside this group provide exciting opportunities for advancing biological insights into processes like photosynthesis, nitrogen fixation, bacterial cell differentiation, and secondary metabolite production (Shih et al., 2013; Knoot et al., 2017). Additionally, taking advantage of the native physiological processes in some genetically intractable species would enhance the potential of these organisms to serve as bioproduction platforms. Therefore, investigating whether these lesser-studied species can be naturally transformed with the appropriate experimental parameters is key to understanding this unique group of organisms.

In addition to serving as model systems, cyanobacteria are also promising platforms for industrial production. These organisms can be engineered to direct energy harvested from the sun into the synthesis of valuable chemicals. Some cyanobacteria have already been genetically modified to produce useful compounds that serve as biofuels, therapeutics, herbicides, and insecticides. However, many species with the potential to synthesize these products cannot yet be developed for this task; the inability to genetically modify these organisms limits their potential to serve as efficient biological factories. Natural transformability is a desirable attribute for emerging biological chassis because it provides a straightforward method for making the extensive genetic changes that are needed to develop such engineered systems.

Although there are other techniques to introduce foreign DNA into cyanobacteria, natural transformation provides unique advantages over these approaches. Conjugation has been successfully demonstrated in many species of cyanobacteria, and has enabled genetic manipulation in several species of bacteria that are experimentally recalcitrant to other transformation methods (Koksharova and Wolk, 2002). Many species in the unicellular diazotrophic *Cyanothece* genus, for example, have proven impossible to transform despite experimental efforts (Liberton et al., 2019). One drawback of conjugal transformation is that it is contingent upon the successful amplification of plasmid constructs in a donor bacterial strain. Unfortunately, some cyanobacteria-specific genomic sequences, including components of the photosynthetic apparatus, are toxic to the most commonly used donor bacterial strain, *Escherichia coli* (Golden et al., 1986; Nagarajan et al., 2011). With natural transformation, linear PCR fragments can serve as donor DNA, bypassing the need to transform donor DNA into *E. coli* (Kufryk et al., 2002). Additionally, transfection of cyanobacteria by electroporation has drawbacks; past studies have suggested that electroporation is inefficient and requires large amounts of donor DNA (Thiel and Poo, 1989; Toyomizu et al., 2001). Furthermore, the extracellular polysaccharide layers found in some cyanobacterial species are physical barriers for entry of the DNA into the cell (Stucken et al., 2012). A few cyanobacterial species have been experimentally demonstrated to be capable of undergoing transformation via electroporation (Koksharova and Wolk, 2002; Stucken et al., 2012; Tsujimoto et al., 2015). Recent advances in microfluidics technology have allowed electroporation experiments to be optimized on a larger scale than was previously feasible (Madison et al., 2017). This technology may enable genome modification in some cyanobacterial species in the future. Moreover, there are a couple of published instances of success with biolistic transformation (Takeyama et al., 1995; Stucken et al., 2012). However, even with these varied approaches, a relatively small number of cyanobacteria have been proven to be capable of transformation.

The advent of whole genome sequencing technology has provided new opportunities to better understand natural transformation in cyanobacteria. This technology has advanced the field, but there are remaining gaps in knowledge about cyanobacterial biology. Understanding the process of natural transformation in cyanobacteria will catalyze breakthroughs on other aspects of cyanobacterial biology by enabling targeted genetic modification in additional species.

## Previous Work to Identify Naturally Transformable Species

Originally, efforts to understand natural transformation in cyanobacteria focused on identifying strains that could be transformed with naked genomic DNA. From these studies, three species have emerged as model systems that are consistently capable of efficient natural transformation. These species are *Synechococcus* sp. PCC 7002, *Synechococcus elongatus* PCC 7942, and *Synechocystis* sp. PCC 6803 (Koksharova and Wolk, 2002). Even though new strains of cyanobacteria continue to be deposited in culture collections, there have only been a few further reports of successful natural transformation in this group of organisms, including studies in *Nostoc muscorum*, *Thermosynechococcus elongatus* BP-1, *T. vulcanus*, *Microcystis aeruginosa* PCC 7806, and recently, *S. elongatus* PCC 11801 (Trehan and Sinah, 1981; Verma et al., 1990; Dittmann et al., 1997; Onai et al., 2004; Jaiswal et al., 2018). Additionally, the species *S. elongatus* UTEX 2973 has been genetically modified to be naturally transformable (Li et al., 2018). Natural transformability was introduced to *S. elongatus* UTEX 2973 by inserting a constitutively expressed copy of the *pilN* gene encoding a component of the DNA uptake apparatus from the naturally transformable cyanobacterium *S. elongatus* PCC 7942. Although transformation efficiency is quite low in this transgenic line, efforts such as these demonstrate how a genomics approach can be used to successfully restore natural transformability when most of the major genetic components are intact.

The early experiments that identified naturally transformable cyanobacterial species employed a simple, but effective experimental setup. Genomic DNA was isolated from mutant lines that were generated under selective growth conditions. The mutant DNA was subsequently used to transform wild type cells of the same species, and transformants were isolated by cultivating cells on selective medium (Shestakov and Khyen, 1970; Stevens and Porter, 1980; Grigorieva and Shestakov, 1982). Although this method provides valuable information about whether the natural transformation machinery functional in a species, it does not determine whether transformation is possible with DNA that does not originate from a cyanobacterial cell.

In modern practice, it is typical to use donor DNA that is isolated from *E. coli* or synthesized with PCR for natural transformation. Interestingly, one study has demonstrated that exposing donor DNA to indigenous methylases prior to transformation increases efficiency in *Synechocystis* sp. PCC 6803 (Wang et al., 2015). Future efforts to enable natural transformation in other cyanobacterial species with donor DNA that does not originate from a cyanobacterial cell may thus focus on exposing the transforming fragments to the appropriate complement of restriction methylases prior to transformation (Stucken et al., 2013). Additionally, a recent study has demonstrated increased transformation efficiency with linear donor DNA fragments by treating cells with an exonuclease inhibitor (Almeida et al., 2017). With annotated genome sequences readily available, identifying the appropriate suite of restriction modification enzymes is a newly achievable task that may enable genetic modification in species of cyanobacteria that were previously intractable. Future efforts to enable genetic analysis in additional cyanobacterial species may involve designing vectors that do not contain sites that are recognized by the target organism’s restriction enzyme system. Furthermore, amplifying donor DNA in engineered bacterial strains that methylate the construct to resemble the native DNA of the target organism has been successful in the past, and may be key to achieving genetic tractability in the future (Wolk et al., 1984).

## Using Genomics to Better Understand Cyanobacterial Natural Transformation

**FIGURE 1:**
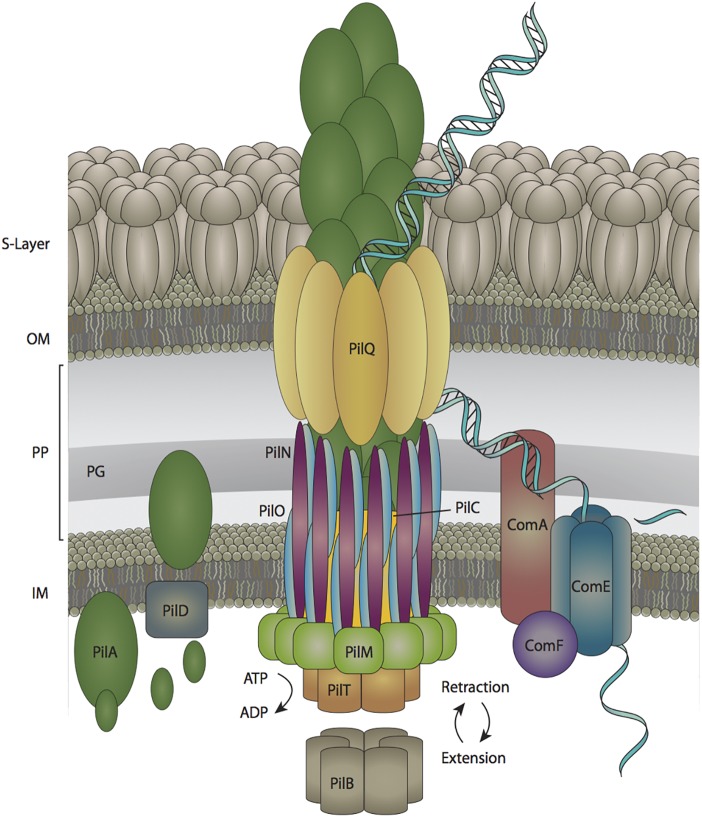
The DNA uptake apparatus in *Synechocystis* sp. PCC 6803. DNA enters the periplasm through an outer membrane pore composed of PilQ subunits. The double stranded DNA is then converted to single-stranded form prior to traversing the inner membrane pore, which is composed of ComE subunits. S-Layer is the surface layer, OM is the outer membrane, PP is the periplasm, PG is the peptidoglycan layer, and IM is the inner membrane.

A Type IV-like pilus facilitates the DNA-uptake step of natural transformation in cyanobacteria and this structure has been well-characterized in other naturally transformable Gram-negative bacteria (Johnsborg et al., 2007). The small number of mutational studies that have been performed on components of the pilus apparatus in cyanobacteria show that the transformation pilus and motility pilus share some of the same protein components in the cyanobacterium *Synechocystis* sp. PCC 6803 (Schuergers and Wilde, 2015). Applying the knowledge that has been gained through more extensive studies in other species to cyanobacterial genomic and experimental data, a preliminary model of the cyanobacterial transformation pilus can be compiled, as shown in Figure 1.

The transformation pilus is a retractable appendage comprised of PilA monomers (Bhaya et al., 2000). Prior to polymerization, PilA monomers are modified by the PilD protein, which cleaves off a leader sequence from pre-pilin PilA monomers (Nunn and Lory, 1991; Bhaya et al., 2000). The motor proteins PilB and PilT are used to extend and retract the arm, respectively, via polymerizing and depolymerizing PilA subunits (Yoshihara et al., 2001; Okamoto and Ohmori, 2002; Nakasugi et al., 2007). The outer membrane pore through which the pilus extends is composed of PilQ monomers and the inner membrane pilus anchor is a combination of PilC, PilM, PilN, and PilO subunits (Bhaya et al., 2000; Yoshihara et al., 2001; Chen, 2007). Additionally, there are proteins that facilitate the transfer of DNA through the inner membrane into the cytoplasm including ComA, ComE, and ComF (Yoshihara et al., 2001; Nakasugi et al., 2006). Moreover, there are several other proteins in *Synechocystis* sp. PCC 6803 (PilJ, PilL-C, and Hfq) that are involved in natural transformation, but their roles in this process are poorly defined (Yoshihara et al., 2002; Dienst et al., 2008).

Using this model for the transformation pilus, we proceeded to analyze 345 cyanobacterial genomes to determine the prevalence of individual protein components across the genus as seen in Table 1. As expected, the species of cyanobacteria known to be naturally competent appear to have a complete set of the known transformation pilus structural and assembly genes, displayed in Supplementary Table S1. Admittedly, there may be other genes that are essential to DNA-uptake in cyanobacteria that have not yet been identified and thus could not be included in this analysis. Overall, this analysis suggests that around seventy percent of species have a complete set of the genes needed to transfer DNA through the outer membrane. Furthermore, one recent genomics study in cyanobacteria has cataloged the genes that are involved in natural transformation post entry of the DNA into the periplasm in a similar manner (Cassier-Chauvat et al., 2016). The data from the genomic analysis of components involved in the transfer of DNA from the periplasm to the cytoplasm have also been integrated into Table 1 and Supplementary Table S1.

**Table 1:** Annotation-based natural transformation gene analysis of cyanobacterial genomes suggests that the majority of species have at least one copy of each of the genes known to be necessary for transport of extracellular DNA into the cytoplasm.

	*pilA*	*pilD*	*pilB*	*pilT*	*pilC*	*pilM*	*pilN*	*pilO*	*pilQ*	*comA, comE, comF*
	Pre-Pilin Subunit	Pre-Pilin Peptidase	Pilus ATPase		Pilus Assembly Protein	Structural Proteins for Aligning Pilus with Outer Membrane Pore			Outer Membrane Pore Subunit	Complement of Periplasm to Cytoplasm Transport Genes in Chauvat-Cassier 2016 Study
Pfam Identifier	pfam16734	pfam01478	pfam05157	pfam00437	pfam00482	pfam11104	pfam05137	pfam04350	pfam00263	–
KEGG ID	K02650	K02654	K02652	K02669	K02653	K02662	K02663	K02664	K02666	–
Percentage of species with ≥1 copy	70%	83%	81%	82%	82%	69%	75%	72%	79%	80%


*Percentages were calculated by taking the number of species that were found to have at least one copy of a gene and dividing by the number of genomes analyzed (345). Assessments were made based on Pfam and KEGG Orthology assignments on the JGI IMG/M ER Database. If a gene was identified by either tool, it was included in the analysis. Detailed species by species analysis is shown in Supplementary Table S1. Data from the Chauvat-Cassier 2016 study (Cassier-Chauvat et al., 2016) in which 74 genomes overlapped with this study, were incorporated into the rightmost column. Only organisms with *comA*, *comE*, and *comF* present were included in the percentage calculation.*

Interestingly, there were anomalies in the genomic analysis that are worth mentioning. For instance, cyanobacteria are gram-negative, but the ComA protein more closely resembles the version of the protein found in gram-positive bacteria. In gram-negative bacteria, this protein is typically soluble as opposed to being membrane-anchored (Chen and Dubnau, 2004). Additionally, PilP, which appears to be essential for natural transformation in other gram-negative bacteria, is absent across all of the cyanobacterial species that were analyzed. In the future, identifying whether there is a substitute protein that serves the same functional role or determining that cyanobacteria simply do not need a substitute will be an interesting endeavor.

Efforts to experimentally transform most of the organisms included in this analysis are yet to be performed. But these data suggest that many of these organisms have all of the genetic machinery that is known to be necessary for natural transformation. If natural transformation becomes possible for these lesser-studied species, there will be many opportunities for novel scientific investigations. However, if the transformation pilus is non-functional in these species, it poses an interesting evolutionary question for why these numerous genes have been preserved over millennia. In either case, experimental analysis will provide further insight on natural transformation in cyanobacteria.

Although there may be other components in this apparatus, they have not yet been characterized. The difficulty of designing an appropriate screen or selection for natural transformability may be to blame for the open-ended structure of this apparatus. However, new screening technology such as random bar code-transposon site sequencing, which does not require screening individual mutant lines for a specific phenotype (Wetmore et al., 2015) may be helpful. This technology has already been applied to screen for genes in other pathways in the naturally transformable cyanobacterium *S. elongatus* PCC 7942, and may help identify other genes involved in cyanobacterial natural transformation as well (Rubin et al., 2015).

## Future Outlook for Understanding Natural Transformation in Cyanobacteria

Natural transformation was the first method that was used to genetically modify cyanobacterial species in the laboratory. However, this process has remained remarkably uncharacterized as other genetic modification techniques have become available in a handful of species. Many members of the cyanobacterial research community still rely heavily on natural transformation to generate mutant strains in an efficient manner. Furthermore, it is crucial that this process be better-characterized in cyanobacteria to take full advantage of the potential for these organisms to serve as bioproduction platforms.

Advances in transformation techniques other than natural transformation have unlocked new opportunities to introduce natural transformability into some cyanobacterial species. Using transformation methods that are established combined with genomics analysis, components of the restriction modification system that serve as barriers to natural transformation can be inactivated. Additionally, it might be possible to replace missing components of the transformation pilus; a substitute gene in a closely related cyanobacterium could be integrated in *trans* to enable natural transformation. Although this approach would not necessarily enable genetic manipulation in species that are currently recalcitrant, it would allow for transformation with donor DNA that is linear and unmethylated. Since the field is nearing a time when it will be more commonplace to order a genome-targeting fragment instead of synthesizing it in the laboratory, the ability to undergo natural transformation is becoming increasingly important.

Without genetic modification tools, advances in understanding cellular processes that are distinct to certain genetically intractable cyanobacterial species have been impeded. Recent innovations in genomic technology have opened up exciting opportunities to better understand this process. The ability to accurately identify elements of restriction modification systems by analyzing sequencing data provides opportunities to appropriately treat donor DNA prior to transformation. Low cost genome sequencing and new analysis tools allow for efficient genomic assessment to determine whether it is worthwhile to attempt experimental optimization for natural transformation on a particular species. New methodologies for genetic screening enable identification of natural transformation proteins without the need to individually assess each mutant line. No doubt as new genomic technologies are developed, advancements in understanding natural transformation in cyanobacteria will soon follow. But in order to get there, the cyanobacteria research community will need to prioritize the development of genetic manipulation systems for these fascinating and diverse organisms.

## Data Availability

Publicly available datasets were analyzed in this study. This data can be found here: https://img.jgi.doe.gov/.

## Author Contributions

KW performed the genomic analysis. KW and HP wrote the manuscript.

## Conflict of Interest Statement

The authors declare that the research was conducted in the absence of any commercial or financial relationships that could be construed as a potential conflict of interest.
